# CB2 Receptors and Neuron–Glia Interactions Modulate Neurotoxicity Generated by MAGL Inhibition

**DOI:** 10.3390/biom10081198

**Published:** 2020-08-18

**Authors:** Estefania Rojo-Bustamante, Ignacio Íñigo-Marco, Miguel Angel Abellanas, Rodrigo Vinueza-Gavilanes, Ana Baltanás, Esther Luquin, Montserrat Arrasate, Maria S. Aymerich

**Affiliations:** 1 Facultad de Ciencias, Departamento de Bioquímica y Genética, Universidad de Navarra, 31008 Pamplona, Spain; erojo@umanizales.edu.co (E.R.-B.); mabellanas.1@alumni.unav.es (M.A.A.); 2 CIMA, Programa de Neurociencias, Universidad de Navarra, 31008 Pamplona, Spain; iinigo@alumni.unav.es (I.Í.-M.); rvinueza@alumni.unav.es (R.V.-G.); abaltana@unav.es (A.B.); marrasatei@unav.es (M.A.); 3 Facultad de Medicina, Departamento de Patología, Anatomía y Fisiología, Universidad de Navarra, 31008 Pamplona, Spain; meluquin@unav.es; 4 IdiSNA, Instituto de Investigación Sanitaria de Navarra, 31008 Pamplona, Spain

**Keywords:** monoacylglycerol lipase, endocannabinoid system, CB2 receptors, microglia, neuroprotection, KML29

## Abstract

Monoacylglycerol lipase inhibition (MAGL) has emerged as an interesting therapeutic target for neurodegenerative disease treatment due to its ability to modulate the endocannabinoid system and to prevent the production of proinflammatory mediators. To obtain a beneficial response, it is necessary to understand how this inhibition affects the neuron–glia crosstalk and neuron viability. In this study, the effect of MAGL inhibition by KML29 was evaluated in two types of rat cortical primary cultures; mixed cultures, including neuron and glial cells, and neuron-enriched cultures. The risk of neuronal death was estimated by longitudinal survival analysis. The spontaneous neuronal risk of death in culture was higher in the absence of glial cells, a process that was enhanced by KML29 addition. In contrast, neuronal survival was not compromised by MAGL inhibition in the presence of glial cells. Blockade of cannabinoid type 2 (CB2) receptors expressed mainly by microglial cells did not affect the spontaneous neuronal death risk but decreased neuronal survival when KML29 was added. Modulation of cannabinoid type 1 (CB1) receptors did not affect neuronal survival. Our results show that neuron–glia interactions are essential for neuronal survival. CB2 receptors play a key role in these protective interactions when neurons are exposed to toxic conditions.

## 1. Introduction

The lipid messenger 2-arachidonoyl glycerol (2-AG) is an important regulator of inter-neuronal and neuron–glia interactions [1,2,3]. In 1995, it was identified as an endogenous cannabinoid receptor ligand [4] that, in the nervous system, it is synthesized on demand by neuronal and non-neuronal cells [5,6]. Additionally, 2-AG signaling impacts on diverse neurophysiological processes that include locomotor activity, learning and memory, pain sensation, stress and anxiety or inflammation among others [7]. It behaves as a full agonist of cannabinoid receptors type 1 (CB1) and type 2 (CB2) [8,9,10]. The CB1 receptor is the most highly expressed G protein-coupled receptor in the brain [11,12]. Conversely, the expression of CB2 receptors in central neurons is absent or very low under normal conditions and it is upregulated during neurodegenerative and neuroinflammatory diseases [13,14]. The selective presence of CB2 receptors on microglial cells is in line with the anti-inflammatory effect of CB2 receptor activation [15,16,17,18]. To decrease 2-AG levels and signaling, the ester linkage between arachidonic acid and glycerol is hydrolyzed [19]. Monoacylglycerol lipase (MAGL) is a ubiquitously expressed serine hydrolase responsible for 85% of 2-AG hydrolysis in the mouse brain [20]. The tissue specificity of MAGL has been further investigated using the MAGL inhibitor JZL184 and MAGL knock-out mice, showing a prominent increase of 2-AG levels in the brain which is less evident in peripheral tissues [21,22]. In addition, 2-AG catabolism provides a major source of arachidonic acid for the generation of inflammatory eicosanoids in the brain [23].

Direct activation of cannabinoid receptors with *Cannabis sativa* preparations or synthetic cannabinoids exerts beneficial effects on pain, spasticity, appetite and nausea [24]. However, their broad use is constrained by dose-limiting effects. In this context, inhibition of endocannabinoid degradation is emerging as a promising strategy both, to increase cannabinoid signaling and to suppress neuroinflammatory events. Pharmacological MAGL inhibition leads to antinociceptive effects in various models of pain [25,26,27,28,29], anxiolytic-like responses [30], improvement of chronic-stress-induced depression-like behavior [31] and amelioration of stress [32] in a CB1 receptor-dependent manner. However, chronic MAGL inhibition may cause CB1 receptor desensitization [33]. The effect of MAGL inhibition on glial cells seems to affect the inflammatory response. Pharmacological or genetic inactivation of MAGL blocked lipopolysaccharide-dependent microglial activation in mouse models [23,34]. JZL184 improved behavior in models of traumatic brain injury and Parkinson’s disease by mechanisms that involved the release of neurotrophic factors and prevented the inflammatory response [23,35,36]. In contrast, a neuroinflammatory phenotype that correlated with motor coordination impairment was observed in the cerebellum but not in the hippocampus after JZL184 administration [37]. Thus, the consequences of MAGL inhibition on neuron–glia interactions are not clear.

The bidirectional interaction between neurons and glial cells through the release of soluble factors or the formation of ligand-receptor partnerships is essential for normal brain function. Modulation of neuron–glia crosstalk would be highly relevant for neuronal survival when brain homeostasis is lost. The differential location of CB1 and CB2 receptors preferentially expressed in neurons and microglial cells, respectively, suggests that the endocannabinoid system may participate in the neuron–glia communication and the neuronal viability. Thus, we hypothesize that modulation of the endocannabinoid system has consequences on neuronal survival. Previously, our group demonstrated that the neuroprotective effect of JZL184 against 1-methyl-4-phenylpyridinium (MPP^+^) induced neurotoxicity in the SH-SY5Y cell line was mediated by CB2 receptors [38]. However, under these experimental conditions, both cannabinoid receptors were expressed by the same cell type. In this study, we address the effect of MAGL inhibition in a more physiological system in which cannabinoid receptors are segregated, CB1 mainly in neurons and CB2 mainly in glial cells, using rat cortical primary cultures. The effect of MAGL inhibition on neuronal survival, in the presence or absence of glial cells, was evaluated taking advantage of a methodological approach that enables to perform longitudinal survival analysis in neurons expressing fluorescent proteins to estimate the risk of neuronal death [39].

## 2. Materials and Methods

### 2.1. Rat Cortical Primary Cultures

Animal handling was carried out according to the European Community Council Directive (2010/63/EC) and Spanish legislation (Real Decreto 53/2013); ethical protocols were approved by the Ethics Committee of the University of Navarra (051-13 and 038-18). Primary cultures of cortical neurons were established from embryonic days 19–20 Sprague–Dawley rat embryos (Charles River Laboratories, Ecully, France) as previously described [40]. Specifically, embryonic brain cortices were isolated and dissociated with 1.5% papain (Worthington, Lakewood, NJ, USA) for 20 min at 37 °C. Tissue was treated for 20 min with 15 mg/mL trypsin inhibitor (Sigma St. Louis, MO, USA) to inhibit papain. Individual neurons were obtained by mechanical dissociation in Opti-MEM medium (Gibco, Waltham, MA, USA) supplemented with 0.8% glucose (Sigma), 2 mg/mL gentamicin (Gibco) and 0.25 μg/mL fungizone (Gibco). Then, neurons were plated at a density of 5 × 10^5^ per well in prewarmed (37 °C) 24-well plates (Corning Costar, Corning, NY, USA) coated with 5 μg/mL laminin (BD Biosciences, Franklin Lakes, NJ, USA) and 50 μg/mL poly-D-lysine (Millipore, Burlington, MA, USA) and incubated 1 h at 37 °C. Once neurons were attached to the bottom of the wells, Opti-MEM medium was replaced by Neurobasal medium (Gibco) supplemented with 5% fetal bovine serum (FBS) (Hyclone, Logan UT, USA), 1% GlutaMAX (Gibco), 2 mg/mL gentamicin (Gibco) and 0.25 μg/mL fungizone (Gibco). To obtain neuron-enriched cultures, primary cultures were treated with 10 μM AraC (Sigma) dissolved in saline solution (0.9% NaCl) at day in vitro (DIV) 2 for 48 h at 37 °C in 5% CO_2_. Otherwise, neurons remained in the incubator until transfection. For immunofluorescence experiments neurons were plated in 24-well plates with glass coverslips (Thermo Scientific, Waltham, MA, USA) coated with 5 μg/mL laminin and 50 μg/mL poly-D-lysine.

### 2.2. Lipofectamine Transfection of Rat Cortical Primary Cultures and Drug Treatments

At DIV5, mixed rat cortical primary cultures (-AraC) or neuron-enriched (+AraC), were transfected with plasmids expressing green fluorescent protein (GFP) or mCherry fluorescent proteins (pCAGGs-GFP, pCAGGs-mCherry plasmid [40]) using lipofectamine 2000 transfection reagent (Invitrogen Carlsbad, CA, USA). One hour before transfection, the culture medium was replaced with Neurobasal without FBS. For lipofectamine complex formation, the plasmids (1 μg/well of 24-well plate) and the lipofectamine reagent were diluted in Opti-MEM medium and mixed in equal volumes. After incubating at room temperature (RT) for 20 min, 50 μL of the mixture were added to each well and plates were incubated for 2 h at 37 °C. Then cells were washed and the medium was replaced by Neurobasal supplemented with 1% FBS, 1% GlutaMAX (Gibco), 2% B27 supplement (Gibco), 2 mg/mL gentamicin and 0.25 mg/mL fungizone. KML29 250 nM (Cayman Chemical, Ann Arbor, MI, USA) was dissolved in 1.5% DMSO, 5% PEG, 5% TWEEN 80 and added starting at DIV6 until DIV12 every 48 h. Rimonabant 200 nM (Cayman Chemical, Ann Arbor, MI, USA) and AM630 1 μM (Tocris Bioscience, Bristol, UK) were dissolved in DMSO and added starting at DIV6 until DIV12 every 48 h. The vehicle of each treatment was added as control.

### 2.3. Immunofluorescence

Cells grown in coverslips were fixed 2 days after AraC treatment by replacing the medium with 4% paraformaldehyde (Panreac, Darmstadt, Germany) and 4% sucrose dissolved in phosphate-buffered saline (PBS) for 8 min. Then, cells were washed twice with PBS and kept at 4 °C until immunostaining. For immunofluorescence, cells were first permeabilized with PBS and 0.1% Tx-100 (PBT) for 20 min at RT and followed by incubation in 1 M glycine (Bio-Rad, Hercules, CA, USA) for 20 min at RT. Nonspecific binding was blocked with 3% goat serum and 3% BSA (Millipore) dissolved in PBT for 1 h. Next, primary antibodies were incubated in blocking solution for 2 h at RT (Table 1). 

After 3 washes in PBS, the corresponding secondary antibody diluted in blocking solution was added, incubated for 2 h at RT and washed again 3 times. Finally, cells were incubated with DAPI (Gibco; 1:50,000 in PBS) for 5 min and washed 3 times with PBS. Coverslips were removed and placed on microscope slides on an 8 μL drop of mounting medium (25 mg of 1,4-diazabicyclo [2.2.2]octane) (DABCO; Sigma) per milliliter of Permafluor (Thermo Scientific, Waltham, MA, US). The preparations were dried for 30 min at 37 °C and kept at 4 °C until they were visualized.

### 2.4. Fluorescence-Activated Cell Sorting

Mixed cultures were plated at a density of 4 × 10^6^ in P100 plates (Corning). At DIV12, cells were collected, washed with PBS and stained for 5 min with Zombie NIR Dye (BioLegend, San Diego, CA, USA; 1:2000 in PBS) at RT. The staining was quenched with flow cytometry buffer, which consisted of PBS supplemented with 0,5% FBS, 1% penicillin/streptomycin (Gibco) and 5 mM EDTA (Invitrogen). Cells were centrifuged at 300× *g* for 5 min and resuspended in 200 µL of flow cytometry buffer that contained a cocktail with the following fluorochrome-conjugated antibodies: anti-CD45 (BioLegend, FITC, 1:200), anti-O4 (Miltenyi Biotec, Bergisch Gladbach, Germany; PE, 1:200) and anti-GLAST (Miltenyi Biotec, APC, 1:100); and were incubated at 4 °C for 15 min. Cells were centrifuged at 300× *g* for 5 min, resuspended in 500 µL of flow cytometry buffer and sorted in a FACS Aria IIu (BD Biosciences). Separated cells were centrifuged at 300× *g* for 5 min, resuspended in 200 µL of thioglycerol/homogenization buffer from the Maxwell RSC simplyRNA Tissue Kit (Promega, Madison, WI, USA) and stored at −80 °C until processing. 

### 2.5. RNA Extraction and PCR Reaction

Cells were plated at a density of 2 × 10^6^ per well in 6-well plates (Corning), at appropriate times the culture medium was replaced by 1 mL of Trizol (Sigma) and RNA was purified following the manufacturer’s instructions, including treatment with 2 U of DNase I (Thermo Fisher Scientific, Waltham, MA, USA), 40 U of Recombinant RNase inhibitor (Takara Bio, Shiga, Japan) and 25 mM EDTA in a final volume of 20 µL. When RNA was extracted from sorted cells, the manufacturer’s instructions were followed in a Maxwell RSC 48 (Promega). Reverse transcription of RNA (2 µg) was performed with 400 U Superscript IV (Thermo Fisher Scientific), 40 U Recombinant RNase inhibitor (Takara Bio Inc., Shiga, Japan) and 5 µM random oligodeoxyribonucleotides hexamers (Thermo Fisher Scientific) in a final volume of 40 µL. RNA expression was assessed in a 2720 thermal cycler (Applied Biosystems, Foster City, CA, USA), 0.5 µL of cDNA, 2.5 U of AmpliTaqGold^®^ DNA polymerase and 0.3 µM forward and reverse primers. Real-Time PCRs were performed with 4.4 µL cDNA (1:5 in water), 0.3 µM forward and reverse primers and 5 µL iQ SYBR Green Supermix (Bio-Rad, Hercules, CA, USA) in a CFX96 Touch real-time detection system (Bio-Rad). The primers sequences were: CB1 receptor (NM_012784.4), forward 5′-TCCCATTTCAAGCAAGGAGCA-3′, reverse 5′-ATTCGAGCCCACGTAGAGGA-3′; CB2 receptor (NM_001164142.3), forward 5′-GGCCACCCAGCAAACATCTA-3′, reverse 5′-TGCTGCGCATCACTCAAGAT; GAPDH (NM_017008.4), forward 5′-AAACCCATCACCATCTTCCA-3′, reverse 5′-GTGGTTCACACCCATCACAA-3′. Real-time PCR results were normalized to GAPDH, and the amount of each transcript was expressed as 2^−ΔCt^ (ΔCt = Ct [GAPDH] − Ct [gene]).

### 2.6. Automated Image Acquisition

Transient transfections with fluorescent-tagged proteins allow us to track individual neurons in culture over long periods of time [39,41]. Neuronal survival was studied by automatic longitudinal tracking of neuronal cultures every 24 h after transfection with GFP on a Zeiss Observer Z1 microscope as previously described [40]. Transfected primary neurons were placed on a Zeiss Observer Z1 microscope that maintains stable conditions for temperature and CO_2_ (37 °C and 5% CO_2)_. Images were acquired automatically at determined positions (designated with particular spatial coordinates) with the 10× long distance objective using the Zen System software (Zeiss). The software enables a sequential and automated repetition of a series of tasks such as locating a particular neuronal field, automatic focusing and image acquisition, and moving forward to the next non overlapping neuronal field. This allows fast and efficient scanning of multiple neuronal fields per plate. Once the full set of images has been acquired, the plate is returned to the incubator until the next scanning. For a typical survival experiment, 10 positions per well and 4 wells per condition were used. Positions were chosen randomly and the selection of neurons to analyze was therefore unbiased. To track the same neuronal fields, a template with the same initial spatial positions was used through the experiment.

### 2.7. Image Processing and Statistics

GraphPad Prism 5 software was used to obtain the graphs. For survival experiments, Matlab-based semi-automated ad hoc programs were developed to estimate the survival times (in hours) of individual neurons in the images acquired. Specific details about how the programs work were previously described [40]. Dead neurons identified along the experiment were categorized as uncensored events. Neurons that survive until the end of the experiment are categorized as censored events. Further survival analysis of the data was performed with STATA 12. The Nelson–Aalen cumulative hazard function was used to estimate and plot cumulative risks of death of different experimental conditions. Differences between groups were analyzed with the log-rank test. In the case of immunofluorescence experiments, 6–10 images per experiment were obtained with a 63× objective on a Zeiss Axiovert 200M fluorescence microscope (Zeiss, Oberkochen, Germany). Image acquisition and processing were performed using MetaMorph Microscopy Automation and Image Analysis Software (Molecular Devices, San Jose, CA, USA). The number of positive cells with respect to the total number of DAPI-stained nuclei or the number of transfected cells with respect to a specific type of cell was calculated. 

## 3. Results

### 3.1. Glial Cells Are Necessary for Neuronal Survival

**Figure 1:**
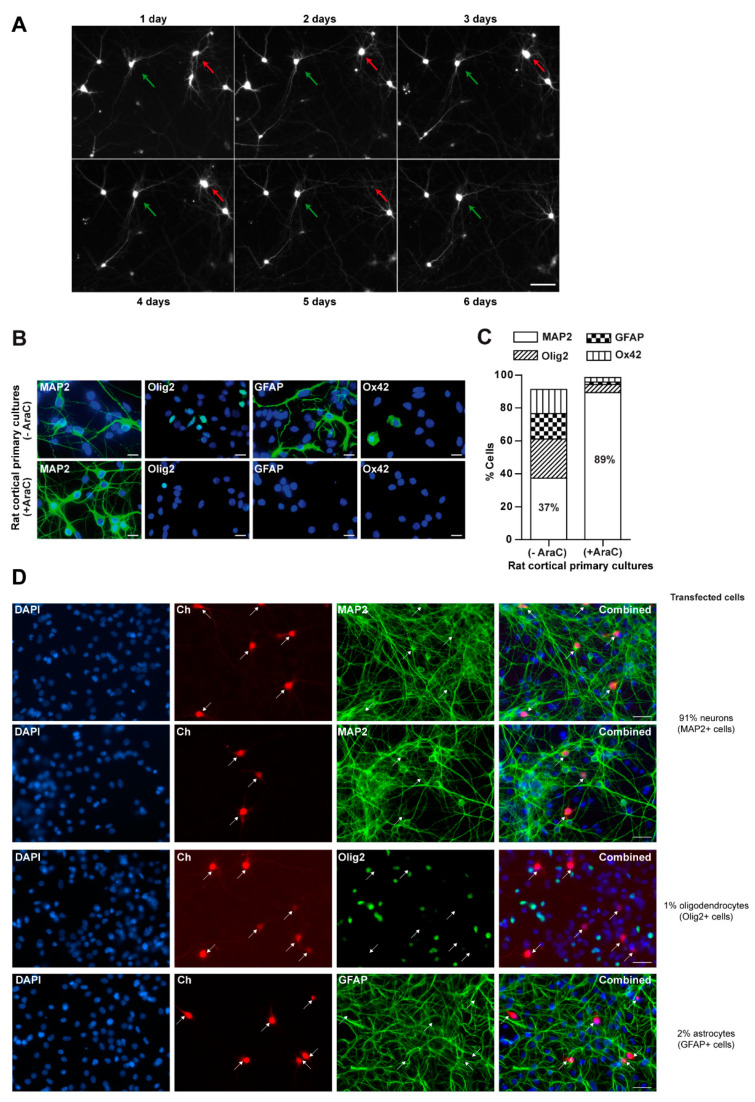
Longitudinal survival analysis in mixed and neuron-enriched cortical primary cultures. (**A**) Example of longitudinal tracking with automated microscopy of individual rat cortical primary neurons transiently transfected with green fluorescent protein (GFP). The green arrow points to a GFP-expressing neuron tracked longitudinally for up to 6 days post-transfection. The red arrow points to another GFP-expressing neuron that survives 4 days post-transfection. Magnification bar: 50 μm. (**B**) Characterization of mixed (−AraC) and neuron-enriched (+AraC) primary cultures. Rat cortical primary cultures were treated with 10 μM AraC at DIV2 for 48 h to prevent glial proliferation. Photomicrographs with representative images corresponding to mixed (upper row) and neuron-enriched (lower row) cultures. The cellular composition of both types of cultures was characterized by immunofluorescence using the following markers: MAP2 for neurons, Olig2 for oligodendrocytes, GFAP for astrocytes and Ox42 for microglia. Cellular nuclei were stained with DAPI. Magnification bars: 10 μm. (**C**) Quantification of the percentage of each cell type relative to the total number of cells (DAPI positive nuclei). Each percentage corresponds to the mean of 3 independent experiments +/- the standard error of the mean: in mixed cultures: MAP2 89 +/− 3.2, GFAP 15 +/− 1.4, Olig2 24 +/− 1.5 and Ox42 15 +/− 1.5; in neuron-enriched cultures: MAP2 89 +/− 3.2, GFAP 1.5 +/− 0.1, Olig2 5 +/− 0.7 and Ox422.8 +/− 1.2 (**D**) Estimation of the percentage of transfected cells that are neurons. Mixed rat primary neuronal cultures were transfected with a plasmid expressing mCherry (Ch). Neurons were fixed 24 h after transfection and immunostained with specific antibodies against MAP2, Olig2 and GFAP. Around 90 transfected cells per condition were analyzed. Magnification bars: 25 μm.

We first evaluated whether glial cells influence neuronal survival by longitudinal survival analysis. This methodological approach in primary neurons requires individual identification and tracking of single neurons. A typical experiment involves transient transfection of primary neurons with plasmids expressing fluorescent proteins such as green fluorescent protein (GFP). Fluorescent neurons are then tracked longitudinally with automated microscopy and the survival time (the last time each neuron was observed alive) is estimated for each individual neuron in the experiment (Figure 1A). Statistics for survival analysis are applied to analyze the data and to quantitatively determine and compare the risk of neuronal death under different experimental conditions [39,40,42].

Here, we compared the risk of death of rat cortical neurons in primary mixed cultures (containing glial cells: astrocytes, oligodendrocytes and microglia) or in enriched neuronal cultures treated with AraC (an inhibitor of glial proliferation). A detailed characterization of mixed and enriched primary cultures was performed using specific antibodies to identify neurons (MAP2), oligodendrocytes (Olig2), astrocytes (GFAP) and microglia (Ox42) by immunofluorescence (Figure 1B). Quantification of the percentage of each cellular type revealed an enrichment of neurons in primary cultures treated with AraC (Figure 1C). Furthermore, the efficiency of neuronal transfection in mixed cultures was analyzed. Immunofluorescence experiments of mixed cultures transfected with a red fluorescent protein mCherry (Ch) indicated that most of the transfected cells were MAP2 positive cells or neurons (around 91%) with a small percentage of glial cells (approximately 2% astrocytes and 1% oligodendrocytes) (Figure 1D). 

**Figure 2:**
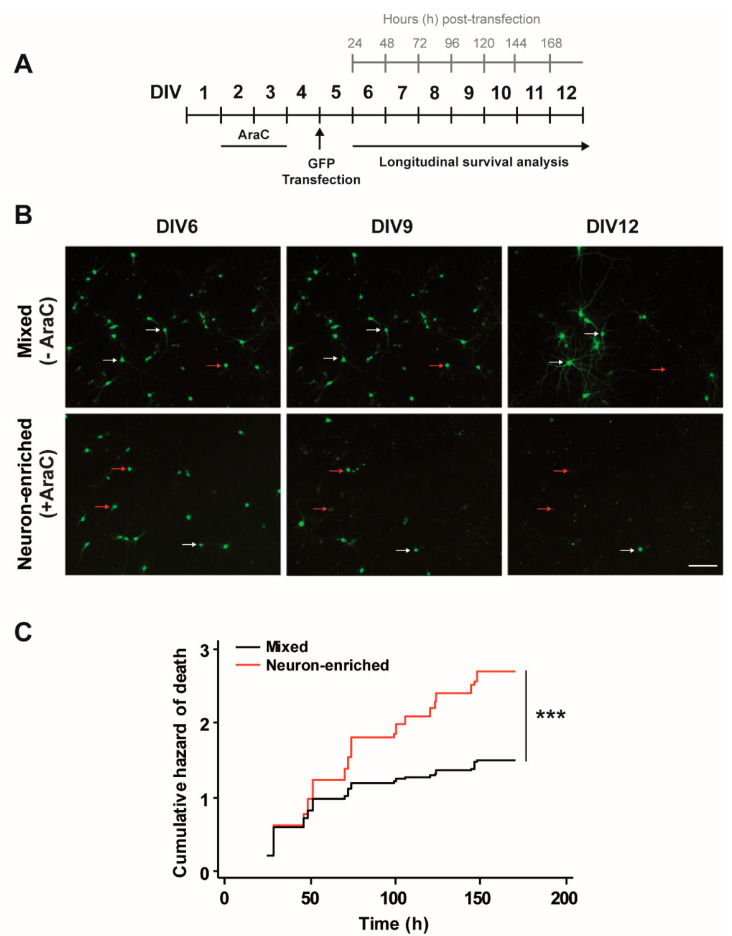
Glial cells are necessary for neuronal survival. (**A**) Diagram showing the experimental design. Rat primary cortical cultures were prepared and 10 μM AraC was added at DIV2 for 48 h to prevent cell proliferation. DIV5 neurons were transfected with GFP and their fate was followed until DIV12. Image acquisition was performed every 24 h after transfection. Red arrows point out to neurons that die along the experimental time whereas white arrows point out to neurons that survive until the end of the experiment. (**B**) Longitudinal monitoring by automated microscopy of individual rat cortical neurons transfected with GFP in the presence and absence of AraC. Magnification bar: 100 μm. (**C**) Cumulative hazard estimates of GFP-transfected neurons from mixed (−AraC) and enriched (+AraC) neuronal cultures. Log-rank test, mixed = 1549 and neuron-enriched = 679 neurons respectively from three independent experiments, *** *p* < 0.001.

Then, GFP-transfected neurons from mixed (−AraC) and enriched cultures (+AraC) were longitudinally tracked with automated microscopy (Figure 2A,B). We observed that neurons from enriched cultures in which glial cells were removed with AraC exhibited a higher risk of death than neurons in mixed cultures (Figure 2C). This result indicates that glial cells present in mixed cultures favor neuronal survival.

### 3.2. Glial Cells Counteract the Neurotoxic Effect of MAGL Inhibition through CB2 Receptors.

**Figure 3:**
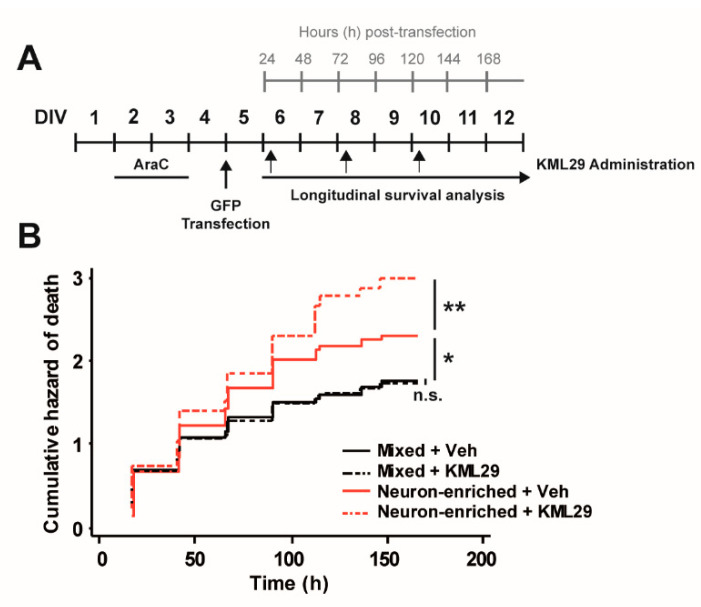
Effect of MAGL inhibition on neuronal survival in the presence or in the absence of glial cells. (**A**) Experimental design: 10 μM AraC was added at DIV2 for 48 h to prevent glial proliferation. Mixed (−AraC) and neuron-enriched (+AraC) cultures were transfected at DIV5 with GFP and treated with KML29 (250 nM) or its vehicle (Veh) starting at DIV6, every 48 h. Longitudinal tracking with automated microscopy of individual rat cortical neurons was performed every 24 h after transfection. (**B**) Cumulative risk estimates of GFP-transfected neurons from mixed (−AraC) and enriched (+AraC) neuronal cultures treated with KML29 or vehicle as control. Log-rank test, *n* = 600–700 neurons per condition from 2 independent experiments, n.s. nonsignificant, * *p* < 0.05, ** *p* < 0.01.

Next, we evaluated the effect of MAGL inhibition on neuronal survival using KML29, a second-generation MAGL inhibitor that shows higher potency (IC_50_ = 43 nM) than JZL184 (IC_50_ = 262 nM) for the rat enzyme inhibition [43]. Starting at DIV6, GFP-transfected neurons from mixed and enriched cultures were treated with 250 nM KML29 every 48 h and subjected to longitudinal survival analysis (Figure 3A). MAGL inhibition significantly increased the risk of death in neurons from enriched primary cultures, but neurons in mixed cultures were not affected. This result strongly suggests that the presence of glial cells counteract the neurotoxic effect of MAGL inhibition with KML29 (Figure 3B). CB1 and CB2 receptors are potential candidates through which this glial-dependent counteractive effect could be mediated. In particular, CB2 receptors that are preferentially expressed in glial cells. 

**Figure 4:**
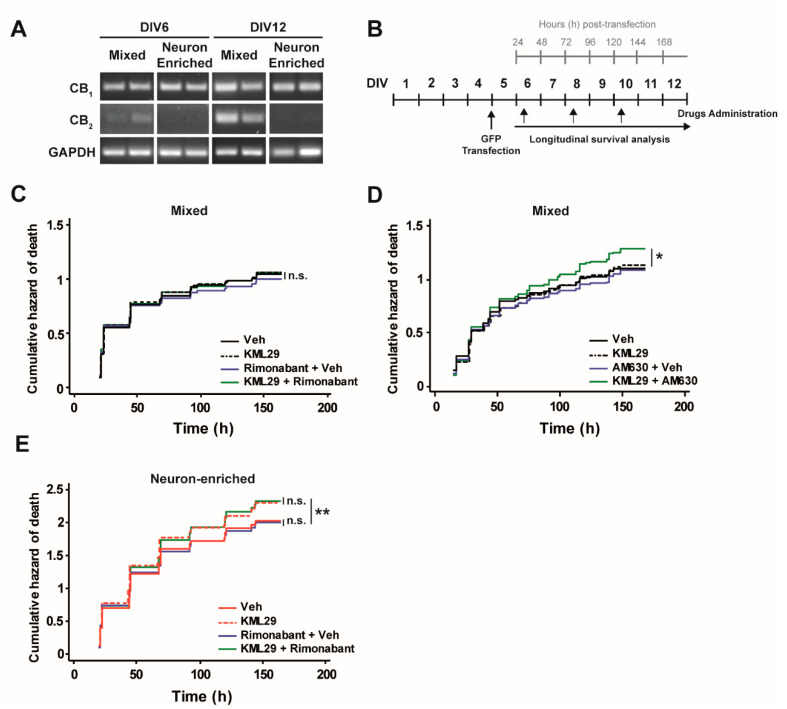
Role of cannabinoid type 1 (CB1) and CB2 receptors blockage on monoacylglycerol lipase inhibition (MAGL) inhibition-dependent neurotoxic effect. (**A**) Analysis of CB1 and CB2 receptors expression by PCR in mixed and neuron-enriched primary cultures at DIV6 and at DIV12 (GAPDH as reference gene). (**B**) Experimental design: 10 μM AraC was added at DIV2 for 48 h to prevent cell proliferation. Mixed (−AraC) and neuron-enriched (+AraC) cultures were transfected at DIV5 with GFP and treated with compounds (250 nM KML29, 200 nM Rimonabant, 1 μM AM630 or vehicle), starting at DIV6 every 48 h. Longitudinal tracking using automated microscopy of individual rat cortical neurons was performed every 24 h after transfection. (**C**) Cumulative hazard estimates of GFP-transfected neurons from mixed (−AraC) neuronal cultures treated with KML29, Rimonabant or both (vehicle as control) (Log-rank test *n* = 650–950 neurons per condition from 3 independent experiments. (**D**) Cumulative hazard estimates of GFP-transfected neurons from mixed (−AraC) neuronal cultures treated with KML29, AM630 or both (vehicle as control). Log-rank test, *n* = 530–840 neurons per condition from 4 independent experiments. (**E**) Cumulative hazard estimates of GFP-transfected neurons from enriched neuronal cultures (−AraC) treated with KML29, Rimonabant or both (vehicle as control). Log-rank test, *n* = 1170–1550 neurons per condition from 3 independent experiments.

Thus, we asked whether the activation of CB1 and CB2 receptors was involved in the glial-dependent protective effect against MAGL inhibition by KML29. First, we analyzed the mRNA expression of CB1 and CB2 receptors by PCR in mixed and enriched neuronal cultures at DIV6 and DIV12. In accordance with its cellular expression pattern, CB1 receptor transcripts were present in both types of cultures. By contrast, CB2 receptor mRNA expression gradually increased in mixed cultures from DIV6 to DIV12 but was not present in neuronal-enriched cultures (Figure 4A). We attributed the increase in CB2 receptor expression to the proliferation of glial cells overtime. To further determine the involvement of these receptors in the glial-dependent protective effect against MAGL inhibition, GFP-transfected neurons from mixed and enriched primary cultures were treated with Rimonabant, a CB1 receptor antagonist, or AM630, a CB2 receptor antagonist, and subjected to longitudinal survival analysis (Figure 4B). In mixed cultures, the risk of neuronal death was not affected by the concomitant use of KML29 and Rimonabant (Figure 4C). Interestingly, when mixed cultures were treated with KML29 and AM630, the risk of neuronal death was significantly increased (Figure 4D). Treatment with only AM630 did not affect neuronal survival. This result strongly suggests that the glial-dependent protective effect against MAGL inhibition seems to be mediated by CB2 receptor activation. To evaluate the potential involvement of CB1 receptors in KML29-dependent neurotoxicity of enriched neuronal cultures, treatments with Rimonabant were performed. However, Rimonabant did not enhance KML29-dependent neurotoxicity (Figure 4E). Altogether, these results suggest that the activation of CB2 receptors in glial cells plays a key protective role in the neuronal survival against the toxicity induced by MAGL inhibition. 

**Figure 5:**
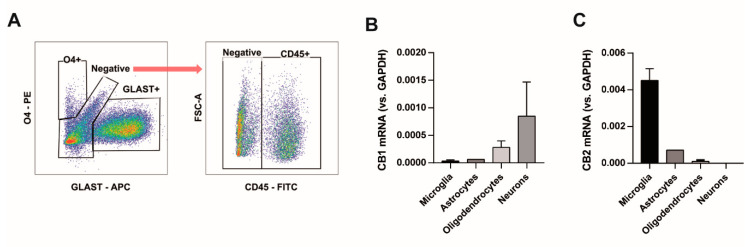
CB2 receptors are expressed by microglial cells at DIV12. Cells from mixed rat cortical primary cultures were collected and immunolabeled to separate different cell types. (**A**) Representative dot plot of fluorescence-activated cell sorting of cells based on the surface expression of O4 (mature oligodendrocytes), GLAST (astrocytes) and CD45 (microglial cells). The negative fraction was enriched in neuronal cells. (**B**) Relative CB1 receptor mRNA expression by real-time PCR in sorted cells. (**C**) Relative CB2 receptor mRNA expression by real-time PCR in sorted cells. PCR reactions were performed in triplicates from 2 independent experiments. Mean +/− standard error of the mean is shown.

To determine which cell type contributed to the CB2 receptor protective effect, cells from mixed cultures were collected at DIV12 and separated by flow cytometry based on the cell surface expression of specific molecules. Mature oligodendrocytes were selected as the O4^+^ population, astrocytes were the GLAST^+^ cells and microglia CD45^+^ (Figure 5A). The remaining cells contained mainly neurons and oligodendrocyte precursors. The expression of CB1 receptors was located in neurons and to a lesser extent in oligodendrocytes (Figure 5B). CB2 receptor expression was predominant in microglial cells and almost undetectable in oligodendrocytes and neurons (Figure 5B), indicating that microglia are key to establish protective neuron–glia interactions.

## 4. Discussion

Our group demonstrated that the neuroprotective effect of JZL184 against MPP^+^-induced neurotoxicity in a neuroblastoma cell line was mediated by CB2 receptors [38]. In this study, we questioned the effect of MAGL inhibition in a system in which cannabinoid receptors are segregated, CB1 mainly in neurons and CB2 mainly in glial cells. To address this question, we used two types of rat cortical primary cultures: mixed cultures that contain non dividing neurons with a spontaneous death rate together with glial cells that divide until they reach confluence; and neuron-enriched cultures that were obtained by eliminating dividing cells with an AraC treatment. We took advantage of a methodological approach that allows to perform longitudinal survival analysis to estimate the risk of neuronal death in the two types of primary cultures. We found that neuronal survival was highly dependent on glial cells, since the spontaneous risk of neuronal death was higher in neuronal-enriched cultures than in mixed cultures. Moreover, MAGL inhibition by KML29 further increased the risk of neuronal death, an effect not observed when glial cells were present in the culture. Interestingly, KML29-dependent toxicity increased in mixed cultures upon blockage of CB2 receptors, indicating that these receptors, expressed mainly in microglial cells, are key players for the protective neuron–glia interactions. Due to this specific location together with their upregulation under pathological conditions [44], our results strongly suggest that CB2 receptors exert selective control over specific neuronal mechanisms that promote neuronal protection and survival. On the contrary, the modulation of CB1 receptors did not affect neuronal survival. 

To determine the effect of MAGL inhibition on neuronal survival, we have used a methodological approach based on automated microscopy to track individual primary neurons expressing a fluorescent protein over long periods of time. This methodology has been applied to estimate the risk of death of neurons overexpressing wild type or mutated forms of proteins associated with neurodegenerative diseases [39,40,41,42]. In this study, we applied this powerful approach to evaluate the effect of glial cells and pharmacological modulators of the endocannabinoid system in neuronal survival. The main advantage of this method over traditional assays is the possibility of following the same neuron over a specific period of time, instead of analyzing different neurons at different time points. This procedure allows the identification of risk factors compromising neuronal survival that otherwise, using conventional methodologies could not be revealed. In fact, no differences were detected with the lactate dehydrogenase assay used in our previous study with SH-SY5Y cells [38]. In the case of mixed primary cultures, neurons account for approximately 40% of the total cells, therefore factors affecting specifically neuronal vulnerability would be attenuated when the toxicity of the overall cells in the culture is analyzed.

Here, we have found a differential effect of MAGL inhibition on neuronal survival that depends on neuron–glia interactions. In our previous in vitro study with neuroblastoma cells [38], a unique cell type expressing CB1 and CB2 receptors was present in the culture. MAGL inhibition was protective when cell death was induced with MPP^+^ in a CB2 receptor-mediated manner. In rat cortical primary cultures, CB1 and CB2 receptors are expressed in different cell subsets, neurons and glia, respectively. In this case, the effect of MAGL inhibition was assayed on the basal neuronal survival rate, in the absence of neurotoxic stimuli. Under these conditions, KML29 was neurotoxic for neurons exclusively when glial cells were absent from the culture, suggesting that CB2 receptors could be relevant in promoting neuronal survival when neurons are exposed to damaging insults. The fact that CB2 receptors increase their expression upon microglial activation further supports its role in neuronal survival under pathological conditions. In vivo studies show that MAGL inhibition is neuroprotective and improves motor behavior in different experimental models of Parkinson’s disease, an effect mediated by a decrease in inflammation and glial cells [23,36]. However, genetic and pharmacological blockade of MAGL resulted in a concomitant microglial activation and exacerbated a proinflammatory reaction in the cerebellum, leading to motor impairment. This reaction was not observed in other brain regions [37]. Importantly, the cerebellum presents the lowest glia/neuron ratio, 0.23, compared to the rest of the brain with an estimated ratio of 11.35 [45,46], and this fact could explain the differences observed. In our primary cultures, with a glia/neuron ratio of 1.5 in mixed cultures and 0.01 in neuron-enriched cultures, the inhibition of MAGL was innocuous in the presence of glial cells; the risk of neuronal death of neurons remained stable in the presence of KML29. Altogether, these observations suggest that MAGL might exert a protective effect when neurons are exposed to harmful conditions (i.e., MPTP in the experimental models of PD), but does not affect the spontaneous death rate.

Our results indicate that inhibition of MAGL by KML29 constitutes a neurotoxic insult for neurons that disappears in the presence of glia. The protective effect that glial cells exert over neurons seems to be mediated by CB2 receptors expressed by microglial cells. CB2 receptors are almost undetectable in the healthy brain but their expression is upregulated under pathological conditions [47]. In culture, resting microglial cells acquire an amoeboid shape and become highly proliferative resembling microglia found in injured tissue [48,49]. The increase in CB2 receptor expression over time detected in the mixed cultures would reflect this activated state of microglial cells. Under standard culture conditions, the risk of neuronal death was unaffected by CB2 receptor blockage with AM630. However, these receptors were necessary to prevent the toxic effect that KML29 caused directly to neurons. Possible mechanisms for this effect would be the switch in microglial phenotypes after CB2 receptor activation with a shift in the gene expression balance towards a neuroprotective phenotype that releases neurotrophic factors and a different pattern of pro- and anti-inflammatory cytokines [36,50]. In addition, endocannabinoids potentiate the expression of CX_3_CR1 [51] and CD200R [52] that interact with their corresponding ligands in the cell surface of neurons promoting the establishment of protective neuron–glia interactions. Modulation of CB1 receptors did not affect neuronal survival at any condition, suggesting a preferential role in other functions not necessarily related to degeneration or repair. These results show the relevant role of microglial cells in protective neuron–glia interactions mediated by CB2 receptors when neurons are exposed to toxic conditions.

Understanding the molecular pathways that cells in the brain use to communicate with each other will shed light on key features of the physiology and pathology of the nervous system. An unmet need for neurodegenerative diseases is the knowledge of molecular pathways that prevent neuronal death under specific neurotoxic stimuli. MAGL inhibition prolongs 2-AG action in the brain through CB1 and CB2 receptors and reduces de biosynthesis of arachidonic acid, the precursor of proinflammatory prostanoids, for these reasons it has emerged as an interesting target for the treatment of neurodegenerative diseases [53]. In this study, we show that MAGL inhibition is toxic for neurons but glial cells counteract this neuronal damage. No degeneration has been reported when MAGL inhibitors are administered in vivo, probably because of the neuron–glia interactions previously established. Under in vitro conditions, microglial cells representing 15% of the total cells are the main cell type expressing CB2 receptors. Activation of this receptor prevents neuronal death, in this case, caused by KML29 toxicity. In summary, our results demonstrate that neuronal survival depends on successful crosstalk established between neurons and microglia. CB2 receptors play a relevant role in this type of interactions and consequently in neuronal fate.

## Figures and Tables

**Table 1:** Primary and secondary antibodies.

Antibody	Provider (Catalog Number)	Type	Dilution
MAP2	Sigma (M1406) Clon AP20	Primary mouse monoclonal	1:1000
GFAP	Sigma (G3893)	Primary mouse monoclonal	1:1000
Olig2	Merck-Millipore (MABN50, clon 211F1.1)	Primary mouse monoclonal	1:200
Ox-42	BD Pharmingen (550299)	Primary mouse monoclonal	1:50
Cy5-conjugated goat anti mouse IgG	Jackson Immunoresearch (115-175-166)	Secondary goat Cy5-conjugated	1:500

## Abbreviations

2-AG	2-arachidonoylglycerol
AraC	Cytosine β-D-Arabinofuranoside hydrochloride
CB1 receptor	Cannabinoid receptor type 1
CB2 receptor	Cannabinoid receptor type 2
CD45	Cluster of differentiation molecule 45
GFP	Green fluorescent protein
GLAST	Glutamate-aspartate transporter
MAGL	Monoacylglycerol lipase
MPP^+^	1-methyl-4-phenylpyridinium
